# Supplementary material for: Successional Categorization of European Hemi-boreal Forest Tree Species
Source: Plants (Basel). 2020 Oct 16;9(10):1381. doi: 10.3390/plants9101381 (PMC7603053; doi:10.3390/plants9101381)
Supplement: Supplementary file 1 [file plants-09-01381-s001.pdf]

**Supplemental Materials 1.**

The tree species of the Lithuanian hemi-boreal forest (Table 1) are Scots pine (*Pinus sylvestris* L.), Norway spruce (*Picea abies* L. Karst), silver birch and downy birch (*Betula pendula* Roth and *B. pubescens* Ehrh.), black alder and grey alder (*Alnus glutinosa* L. Gaertn. and *A. incana* L. Moench), white willow and crack willow (*Salix alba* L. and *S. fragilis* L.), Eurasian aspen (*Populus tremula* L.), European ash (*Fraxinus excelsior* L.), English oak (*Quercus robur* L.), small-leaved lime (*Tilia cordata* Mill.), European white elm and wych elm (*Ulmus laevis* Pall. and *U. glabra* Huds.), Norway maple (*Acer platanoides* L.), bird cherry (*Prunus padus* L.), wild apple (*Malus sylvestris* L. Mill.), and wild pear (*Pyrus pyraster* L. Burgsd.); the northern borders of field elm (*Ulmus minor* Mill.), and European hornbeam (*Carpinus betulus* L.) cross Lithuania [1]. European beech (*Fagus sylvatica* L.) could expand its range into the Baltic [2]. Sessile oak (*Quercus petraea* Matt. Liebl.) is supposed to be an introduced species [3]. Wild cherry (*Prunus avium* L.) is spreading to forest stands naturally from domesticated sources [4,5]. Large-leaved lime (*Tilia platyphyllos* Scop.) is not found as a native tree in Lithuania.

**Table 1.** A description of the life history dynamics of hemi-boreal forest tree species and their position in successional categories for Lithuania.

| Tree species | Life history dynamics |
|---|---|
| **Gap colonizers/pioneers** | |
| *Betula pendula* *Betula pubescens* | Silver birch and downy birch are pioneer species that thrive during early stages of secondary vegetation succession [6]. In the later successional forests of the boreal zone of northern Europe, they can dominate [7]. Birches are opportunists in steady-state woodland systems [8]. There is no clear evidence as to their adaptiveness of the environmentally caused variation [9]. |
| *Pinus sylvestris* | Through much of its range Scots pine may be considered post-pioneer to birch and pre-climax to Norway spruce; it is able to colonize nutrient-poor soils after stand-replacing disturbances [10–12]. Scots pine is usually replaced by Norway spruce if there is a lack of fire [13]. It is to be emphasized that the protection of older trees not only promotes well-balanced development of saplings but also gives a necessary impetus to the reproduction [14]. |
| *Populus tremula* | Eurasian aspen is an early clonal pioneer species with fast early growth [15]. A disturbance adapted species and a colonizer of clear disturbed areas such as after fire, clear-cutting, wind-throw, or defoliation are beneficial [16]; it is shade-tolerant and a stable part of a mixed stand woodland, especially with species that allow sunlight through the canopy, such as Scots pine and birches [17]. |
| *Alnus glutinosa* *Alnus incana* | Both species are able to quickly colonize disturbed sites and improve soil conditions for other species [18,19]. Grey alder regenerates vigorously with root suckers, especially in open places [20] and is regarded as a more light-demanding species compared to black alder, which tends to be out-competed by other species once the canopy closes [21]. |
| *Salix alba* *Salix fragilis* | White willow is a fast-growing tree, very tolerant of maritime exposure [22], but is shade intolerant [23]. It is closely allied to crack willow, with which it freely hybridizes [24]. |
| *Prunus avium* *Prunus padus* | Wild cherry and bird cherry colonize early successional stages as a result of forest disturbances [25,26]. It is a seed recruitment pioneer colonization species, which can be followed up by extensive root suckering [27]. Wild cherry seeds easily, germinating under a canopy [28] but prefers full sunlight between the ages of 3 and 5 years [29]. This species has a fast growth rate, which is not paralleled by any other tree species. In open light, wild cherry can outcompete other species but needs a head start of 3–5 years' growth [30]. |
| **Gap competitors—post-pioneers** | |
| *Quercus robur* *Quercus petraea* | Large gaps favor the recruitment of these light-demanding species created by old dying trees, wind, and insects [31–33]. *Q. robur* prefers rich fertile calcareous soils, whereas *Q. peraea* prefers lighter and more acidic soils [34]. In central Europe, their main competitor is beech and other shade or half-shade tolerant trees that block light [35]. Ligot et al. [36] found that beech saplings naturally outcompete oak and ash saplings that have increased light requirement with age. The initial number of plants that survive to form the seedling bank is inversely related to the distance of the nearest parent tree as well as canopy cover [37]. |
| *Fraxinus excelsior* | European ash can be shade tolerant as seedling but in older age are shade intolerant [38]. Young plants show very rapid growth, although full overhead light is necessary for developing vigorous |

| | |
|---|---|
| | trees [39]. Ash is considered an intermediate tree in ecological succession and can take advantage of disturbances at the stand level [40]. |
| *Ulmus laevis* | European white elm is a light-demanding and fast-growing riparian habitat specialist with limited dispersal [41,42]. It can outcompete willows and aspens to become co-dominant with European ash and English oak in the downstream areas of floodplain succession [41]. |
| *Malus sylvestris* | Unlike wild pear, wild apple occurs mostly below the canopy of other broadleaves such as English oak, Eurasian aspen, European ash. Floodplain forests, which are dominated by light-demanding canopy trees, provide favorable conditions for the establishment and reproduction of the wild apple [43]. It prefers permeable clayey soil with shallow ground water [4]. |
| *Pyrus pyraster* | Wild pear prefers fresh calcareous soil for the establishment and growth in gaps or under the see-through canopy of shade-intolerant trees [4]. |
| **Forest colonizers (pre-climax)** | |
| *Picea abies* | Norway spruce, a secondary colonizer, depends largely on advance regeneration to capture small-scale gaps in the mature canopy [44,45]. It is not dependent on the protection of the mother tree; it grows best in depressions under extremely sheltered environmental conditions, where it is immune to all outside influences [14]. The seedlings of Norway spruce can tolerate lateral shade and can outcompete nearly all deciduous trees and ground vegetation [46–49]. |
| *Acer platanoides* | Norway maple germinates and grows rapidly in shade, even under a closed forest canopy [50]; its rapid early growth may cause suppression of the other mixed species [17]. It possesses high density regeneration properties under the canopy near larger gaps [32] and can outcompete nearby plants for nutrient uptake [51–53]. At maturity, it becomes more light-demanding and can co-dominate with other broadleaves such as English oak and small-leaved lime [20,54]. |
| *Ulmus glabra* | Wych elm is found in moist rich forests and in riparian zones with higher, less variable precipitation [55,56]. It is shade tolerant and with Norway maple can outcompete oaks and ashes [57]. In Lithuania, wych elm is mainly found in mature stands dominated by European ash [58]. Formerly, wych elm was the precursor to the climax formation [59]. |
| *Ulmus minor* | Field elm prefers low lying forest along rivers and can tolerate both floods and droughts [56] and is often associated with English oak and European ash [60]. Niinemets and Valladares [61] classified wych elm and field elm as having similar shade tolerance, namely 3.53 and 3.36, respectively, on a scale of 0 (very intolerant) to 5 (very tolerant). Lithuania contains the northern borders of field elm [1]. |
| **Forest competitors (natural climax)** | |
| *Tilia cordata* *Tilia platyphyllos* | Small-leaved lime and large-leaved lime are shade-tolerant and highly competitive [20]. They tend to grow in close proximity to other species in mixed stands [3, 62–64]. In Britain, limes are generally associated with English oak and European beech, and their presence is often taken as an indicator of ancient woodland [17]. |
| *Fagus sylvatica* | European beech is the most shade-tolerant broadleaved tree in its range [39] and the strongest competitor among the trees in its range [65,66]. The high growth rate can remain steady until late maturity [2]. The seedlings are able to tolerate both lateral and vertical shade [47]. In suitable soils and under a closed canopy, very few other species can grow in a beech forest [22]. |
| *Carpinus betulus* | European hornbeam is one of the few strongly shade-tolerant trees [64], though slightly less than beech [17]; it grows mostly in mixed stands below the canopy of other broadleaves such as English oak, European ash, and Norway maple [67]. European hornbeam can be a dangerous invader, regenerating better and faster than English oak, European ash, or Scots pine [39]. It can be grown in mixed stands alongside oaks to produce straighter trees with less epicormic branches [68]. Lithuania contains the northern borders of European hornbeam [1]. |

## References


1. Karazija, S. Types of Lithuanian Forest; Vilnius, Lithuania, 1988. (In Lithuanian)
2. Houston Durrant, T.; de Rigo, D.; Caudullo, G. Fagus sylvatica and Other Beeches in Europe: Distribution, Habitat, Usage and Threats. In European Atlas of Forest Tree Species; San-Miguel-Ayanz, J., de Rigo, D., Caudullo, G., Houston Durrant, T., Mauri, A., Eds.; Publ. Off. EU: Luxembourg, 2016; p. e012b90+.
3. Navasaitis, M.; Ozolinčius, R.; Smaliukas, D.; Balevičienė, J. *Dendroflora of Lithuania*. Lututė: Kaunas, Lithuania, 2003. (In Lithuanian with English summary)

4. Petrokas, R. Phenotypic Variability of Wild Apple and Wild Pear. Ph.D. Thesis, Lithuanian University of Agriculture, Lithuanian Forest Research Institute, Kaunas, Lithuania, 2006.
5. Petrokas, R.; Pliūra, A. Persistence of progenies of wild cherry (*Prunus avium* L.) at northern limit of natural distribution range in transfer to Lithuania. *Balt. For.* **2014**, *20(1)*, 58–69.
6. Beck, P.; Caudullo, G.; de Rigo, D.; Tinner, W. *Betula pendula, Betula pubescens* and other birches in Europe: Distribution, habitat, usage and threats. In *European Atlas of Forest Tree Species*; San-Miguel-Ayanz, J., de Rigo, D., Caudullo, G., Houston Durrant, T., Mauri, A., Eds.; Publ. Off. EU: Luxembourg, 2016; pp. e010226+.
7. Moen, A. *National Atlas of Norway: Vegetation*; Norwegian Mapping Authority: Hønefoss, Norway, 1999; 196 pp.
8. Dickson, J.H. Pleistocene history of *Betula* with special reference to the British Isles. *Proc. R. Soc. Edinburgh* **1984**, *85B*, 1–11. https://doi.org/10.1017/S0269727000003845
9. Davy, A.J.; Gill, J.A. Variation due to environment and heredity in birch transplanted between heath and bog. *New Phytol.* **1984**, *97*, 489–505.
10. Mátyás, C.; Ackzell, L.; Samuel, C.J.A. EUFORGEN Technical Guidelines for genetic conservation and use for Scots pine (*Pinus sylvestris*). International Plant Genetic Resources Institute: Rome, Italy, 2004; 6 pp.
11. Tarasiuk, S.; Zwieniecki, M. Social-structure dynamics in uneven-aged Scots pine (*Pinus sylvestris*) regeneration under canopy at the Kaliszki reserve, Kampinoski national park (Poland). *For. Ecol. Manag.* **1990**, *35*, 277–289.
12. Tansley, A.G. *The British Islands and Their Vegetation*; Volume 1; Cambridge University Press: Cambridge, UK, 1965.
13. Steijlen, I.; Zackrisson, O. Long-term regeneration dynamics and successional trends in a northern Swedish coniferous forest stand. *Can. J. Bot.* **1987**, *65*, 839–848.
14. Schauberger, V. *The Fertile Earth: Nature's Energies in Agriculture, Soil Fertilisation and Forestry*; Coats, C., Ed.; Eco-Technology Series 3; Gill Books: Dublin, Ireland, 2000; 220 pp. Available online: https://alkimist.net/wp-content/uploads/2015/11/Coats-Schauberger-The-fertile-earth-Natures-energies-in-agriculture-soil-fertilisation-and-forestry-2000.pdf (accessed on 20 June 2020).
15. Rogers, P.C.; Pinno, B.D.; Šebesta, J.; Albrectsen, B.R.; Li, G.; Ivanova, N.; Kusbach, A.; Kuuluvainen, T.; Landhäusser, S.M.; Liu, H.; Myking, T.; Pulkkinen, P.; Wen, Z.; Kulakowski, D. A global view of aspen: Conservation science for widespread keystone systems. *Glob. Ecol. Conserv.* **2020**, *21*, e00828. https://doi.org/10.1016/j.gecco.2019.e00828
16. Caudullo, G.; de Rigo, D. *Populus tremula* in Europe: Distribution, habitat, usage and threats. In *European Atlas of Forest Tree Species*; San-Miguel-Ayanz, J., de Rigo, D., Caudullo, G., Houston Durrant, T., Mauri, A., Eds.; Publ. Off. EU: Luxembourg, 2016; pp. e01f148+.
17. Savill, P.S. *The Silviculture of Trees Used in British Forestry*, 3rd ed.; CABI International: Wallingford, UK, 2019; 384 pp.
18. Houston Durrant, T.; de Rigo, D.; Caudullo, G. *Alnus glutinosa* in Europe: Distribution, habitat, usage and threats. In *European Atlas of Forest Tree Species*; San-Miguel-Ayanz, J., de Rigo, D., Caudullo, G., Houston Durrant, T., Mauri, A., Eds.; Publ. Off. EU: Luxembourg, 2016; pp. e01f3c0+.
19. Houston Durrant, T.; de Rigo, D.; Caudullo, G. *Alnus incana* in Europe: Distribution, habitat, usage and threats. In *European Atlas of Forest Tree Species*; San-Miguel-Ayanz, J., de Rigo, D., Caudullo, G., Houston Durrant, T., Mauri, A., Eds.; Publ. Off. EU: Luxembourg, 2016; pp. e01ff87+.
20. Hytteborn, H.; Maslov, A.A.; Nazimova, D.I.; Rysin, L.P. Boreal forests of Eurasia. In *Coniferous Forests*; Andersson, F.A., Ed.; Ecosystems of the World Series 6; Elsevier: Amsterdam, The Netherlands, 2005; pp. 23–99.
21. McVean, D.N. *Alnus glutinosa* (L.) Gaertn. *J. Ecol.* **1953**, *41(2)*, 447–466. https://doi.org/10.2307/2257070
22. Beckett, G.; Beckett, K. *Planting Native Trees and Shrubs*; Jarrold Colour Publications: Norwich, UK, 1979.
23. Houston Durrant, T.; de Rigo, D.; Caudullo, G. *Salix alba* in Europe: Distribution, habitat, usage and threats. In *European Atlas of Forest Tree Species*; San-Miguel-Ayanz, J., de Rigo, D., Caudullo, G., Houston Durrant, T., Mauri, A., Eds.; Publ. Off. EU: Luxembourg, 2016; pp. e01153e+.
24. Bean, W.J. *Trees and Shrubs Hardy in the British Isles*; John Murray: London, UK, 1981.
25. De Rogatis, A.; Ferrazzini, D.; Ducci, F.; Guerri, S.; Carnevale, S.; Belletti, P. Genetic variation in Italian wild cherry (*Prunus avium* L.) as characterized by nSSR markers. *Forestry* **2013**, *86(3)*, 391–400. https://doi.org/10.1093/forestry/cpt009

26. Roon, D.; Wipfli, M.; Wurtz, T.L.; Prakash, A. Distribution of invasive European bird cherry (*Prunus padus*) in riparian forests along urban Alaskan streams. In *Forest Health Conditions in Alaska 2014*; Publication R10-PR-36; USDA Forest Service, Alaska Region, Juneau, AK, USA, 2015; pp. 40–43. Available online: http://www.fs.usda.gov/Internet/FSE_DOCUMENTS/stelprd3834154.pdf (accessed on 3 May 2020).
27. Welk, E.; de Rigo, D.; Caudullo, G. *Prunus avium* in Europe: Distribution, habitat, usage and threats. In *European Atlas of Forest Tree Species*; San-Miguel-Ayanz, J., de Rigo, D., Caudullo, G., Houston Durrant, T., Mauri, A., Eds.; Publ. Off. EU: Luxembourg, 2016; pp. e01491d+.
28. Pryor, S.N. *The Silviculture and Yield of Wild Cherry*; Forestry Commission Bulletin 75; HMSO: London, UK, 1988; pp. 20–21.
29. Vera, F.W.M. *Grazing Ecology and Forest History*; CABI Publishing: Wallingford, UK, 2000; pp. 339–341.
30. Petrokas, R. Prerequisites for the reproduction of wild cherry (*Prunus avium* L.). *Balt. For.* **2010**, *16(1)*, 139–153.
31. Aas, G. *Quercus petraea* (Mattuschka) Lieblein, 1784. In *Enzyklopädie der Holzgewächse. Handbuch und Atlas der Dendrologie*; Roloff, A., Weisgerber, H., Lang, U., Stimm, B., Schütt, P., Eds.; Wiley-VCH: Weinheim, Germany, 2002; pp. 1–16. (In German)
32. Jaloviar, P.; Sedmáková, D.; Pittner, J.; Jarčušková Danková, L.; Kucbel, S.; Sedmák, R.; Saniga, M. Gap structure and regeneration in the mixed old-growth forests of National Nature Reserve Sitno, Slovakia. *Forests* **2020**, *11(1)*, 81. https://doi.org/10.3390/f11010081
33. Götmark, F.; Kiffer, C. Regeneration of oaks (*Quercus robur*/*Q. petraea*) and three other tree species during long-term succession after catastrophic disturbance (windthrow). *Plant Ecol.* **2014**, *215*, 1067–1080. https://doi.org/10.1007/s11258-014-0365-4
34. Stern, K.; Roche, L. *Genetics of Forest Ecosystems*; Ecological Studies Series 6; Springer-Verlag: Berlin Heidelberg New York, 1974. https://doi.org/10.1007/978-3-642-65517-3
35. Eaton, E.; Caudullo, G.; Oliveira, S.; de Rigo, D. *Quercus robur* and *Quercus petraea* in Europe: Distribution, habitat, usage and threats. In *European Atlas of Forest Tree Species*; San-Miguel-Ayanz, J., de Rigo, D., Caudullo, G., Houston Durrant, T., Mauri, A., Eds.; Publ. Off. EU: Luxembourg, 2016; pp. e01c6df+.
36. Ligot, G.; Balandier, P.; Fayolle, A.; Lejeune, P.; Claessens, H. Height competition between *Quercus petraea* and *Fagus sylvatica* natural regeneration in mixed and uneven-aged stands. *For. Ecol. Manag.* **2013**, *304*, 391–398.
37. Harmer, R.; Morgan, G. Development of *Quercus robur* advance regeneration following canopy reduction in an oak woodland. *Forestry* **2007**, *80(2)*, 137–149. https://doi.org/10.1093/forestry/cpm006
38. Beck, P.; Caudullo, G.; Tinner, W.; de Rigo, D. *Fraxinus excelsior* in Europe: Distribution, habitat, usage and threats. In *European Atlas of Forest Tree Species*; San-Miguel-Ayanz, J., de Rigo, D., Caudullo, G., Houston Durrant, T., Mauri, A., Eds.; Publ. Off. EU: Luxembourg, 2016; pp. e0181c0+.
39. Praciak, A.; Pasiecznik, N.; Sheil, D.; van Heist, M.; Sassen, M.; Correia, C.S.; Dixon, C.; Fyson, G.; Rushford, K.; Teeling, C. (Eds.) *The CABI Encyclopedia of Forest Trees*; CABI: Oxfordshire, UK, 2013; 536 pp.
40. Pliūra, A.; Heuertz, M. EUFORGEN Technical Guidelines for genetic conservation and use for common ash (*Fraxinus excelsior*). International Plant Genetic Resources Institute: Rome, Italy, 2003; 6 pp.
41. Marks, C.O. The ecological role of American elm (*Ulmus americana* L.) in floodplain forests of northeastern North America. In Proceedings of the American Elm Restoration Workshop, Lewis Center, OH, USA, 25–27 October 2016; Pinchot, C.C., Knight, K.S., Haugen, L.M., Flower, C.E., Slavicek, J.M., Eds.; Gen. Tech. Rep. NRS-P-174; USDA Forest Service, Northern Research Station, Newtown Square, PA, USA, 2017; pp. 74–98.
42. Vakkari, P.; Rusanen, M.; Kärkkäinen, K. High genetic differentiation in marginal populations of European white elm (*Ulmus laevis*). *Silva Fenn.* **2009**, *43(2)*, 185–196.
43. Schnitzler, A.; Arnold, C.; Cornille, A.; Bachmann, O.; Schnitzler, C. Wild European apple (*Malus sylvestris* (L.) Mill.) population dynamics: Insight from genetics and ecology in the Rhine Valley. Priorities for a future conservation programme. *PLoS ONE* **2014**, *9(5)*, e96596. https://doi.org/10.1371/journal.pone.0096596
44. Caudullo, G.; Tinner, W.; de Rigo, D. *Picea abies* in Europe: Distribution, habitat, usage and threats. In *European Atlas of Forest Tree Species*; San-Miguel-Ayanz, J., de Rigo, D., Caudullo, G., Houston Durrant, T., Mauri, A., Eds.; Publ. Off. EU: Luxembourg, 2016; pp. e012300+.
45. Qinghong, L.; Hytteborn, H. Gap structure, disturbance and regeneration in a primeval *Picea abies* forest. *J. Veg. Sci.* **1991**, *2*, 391–402.

46. Aarrestad, P.A.; Myking, T.; Stabbetorp, O.E.; Tollefsrud, M.M. Foreign Norway spruce (*Picea abies*) provenances in Norway and effects on biodiversity; NINA Report 1075; Foundation Norwegian Institute for Nature Research: Trondheim, Norway, 2014; 39 pp.
47. Stancioiu, P.T.; O'Hara, K.L. Regeneration growth in different light environments of mixed species, multiaged, mountainous forests of Romania. *Eur. J. For. Res.* **2006**, *125*, 151–162. https://doi.org/10.1007/s10342-005-0069-3
48. Tømmerås, B.Å.; Johnsen, Ø.; Skrøppa, T.; Hindar, K.; Holten, J.; Tufto, J. Long-term environmental impacts of release of transgenic Norway spruce (*Picea abies*); NINA NIKU Project Report 003; Foundation for Nature Research and Cultural Heritage Research: Trondheim, Norway, 1996; 48 pp.
49. Jonsson, B.G.; Esseen, P.-A. Treefall disturbance maintains high bryophyte diversity in a boreal spruce forest. *J. Ecol.* **1990**, *78*, 924–936.
50. Caudullo, G.; de Rigo, D. *Acer platanoides* in Europe: Distribution, habitat, usage and threats. In *European Atlas of Forest Tree Species*; San-Miguel-Ayanz, J., de Rigo, D., Caudullo, G., Houston Durrant, T., Mauri, A., Eds.; Publ. Off. EU: Luxembourg, 2016; pp. e019159+.
51. Meiners, S.J. Seed and seedling ecology of *Acer saccharum* and *Acer platanoides*: A contrast between native and exotic congeners. *Northeast. Nat.* **2005**, *12*, 23–32.
52. Philbrick, H.; Gregg, R.B. *Companion Plants and How to Use Them*, 32nd ed.; Floris Books: Edinburgh, UK, 2016.
53. Reinhart, K.O.; Greene, E.; Callaway, R.M. Effects of *Acer platanoides* invasion on understory plant communities and tree regeneration in the northern Rocky Mountains. *Ecography* **2005**, *28*, 573–582. https://doi.org/10.1111/j.2005.0906-7590.04166.x
54. Mitchell, A.F. *A Field Guide to the Trees of Britain and Northern Europe*, 1st ed.; Collins: London, UK, 1974. 415 pp.
55. Thomas, P.A.; Stone, D.; La Porta, N. Biological flora of the British Isles: *Ulmus glabra*. *J. Ecol.* **2018**, *106*, 1724–1766. https://doi.org/10.1111/1365-2745.12994
56. Petrokas, R.; Baliuckas, V. Morphological intergradation of native elm species is shown by site-specific parameters. *Balt. For.* **2014**, 20(2), 238–247.
57. Diekmann, M. Deciduous forest vegetation in Boreo-nemoral Scandinavia. *Acta Phytogeogr. Suec.* **1994**, *80*, 1–112.
58. Petrokas, R. Height growth and its relation to the branching habits of wych elm (*Ulmus glabra* Hudson) in Lithuania. *Balt. For.* **2011**, *17(1)*, 83–90.
59. Sukopp, H.; Trepl, L. Extinction and naturalization of plant species as related to ecosystem structure and function. In *Potentials and Limitations of Ecosystem Analysis*; Schulze, E.-D., Zwölfer, H., Eds.; Ecological Studies Series 61; Springer-Verlag, Berlin, Germany, 1987; pp. 245–276.
60. Heybroek, H.M.; Goudzwaard, L.; Kaljee, H. *Elm, a Tree with Character of the Low Countries*; KNNV Uitgeverij: Zeist, The Netherlands, 2009; 272 pp. (In Dutch)
61. Niinemets, Ü.; Valladares, F. Tolerance to shade, drought, and waterlogging of temperate Northern Hemisphere trees and shrubs. *Ecol. Monogr.* **2006**, *76*, 521–547. https://doi.org/10.1890/0012-9615(2006)076[0521:TTSDAW]2.0.CO;2
62. De Jaegere, T.; Hein, S.; Claessens, H. A review of the characteristics of small-leaved lime (*Tilia cordata* Mill.) and their implications for silviculture in a changing climate. *Forests* **2016**, *7*, 56.
63. Eaton, E.; Caudullo, G.; de Rigo, D. *Tilia cordata*, *Tilia platyphyllos* and other limes in Europe: Distribution, habitat, usage and threats. In *European Atlas of Forest Tree Species*; San-Miguel-Ayanz, J., de Rigo, D., Caudullo, G., Houston Durrant, T., Mauri, A., Eds.; Publ. Off. EU: Luxembourg, 2016; pp. e010ec5+.
64. Spiecker, H.; Hein, S.; Makkonen-Spiecker, K.; Thies, M. (Eds.) Valuable broadleaved forests in Europe; European Forest Institute Research Report 22; Brill: Leiden, The Netherlands, 2009; 276 pp.
65. Ellenberg, H. *Vegetation Mitteleuropas mit den Alpen in Ökologischer Sicht*, 3rd ed.; Ulmer: Stuttgart, Germany, 1982; 998 pp. (In German).
66. Walter, H. *Vegetation of the Earth and Ecological Systems of the Geo-biosphere*, 2nd ed.; Springer-Verlag: New York, NY, USA, 1979; pp. 32–174. https://doi.org/10.1007/978-1-4684-0468-5
67. Kuehne, C.; Nosko, P.; Horwath, T.; Bauhus, J.A. comparative study of physiological and morphological seedling traits associated with shade tolerance in introduced red oak (*Quercus rubra*) and native hardwood tree species in southwestern Germany. *Tree Physiol.* **2014**, *34(2)*, 184–193. https://doi.org/10.1093/treephys/tpt124

68. Sikkema, R.; Caudullo, G.; de Rigo, D. *Carpinus betulus* in Europe: Distribution, habitat, usage and threats. In *European Atlas of Forest Tree Species*; San-Miguel-Ayanz, J., de Rigo, D., Caudullo, G., Houston Durrant, T., Mauri, A., Eds.; Publ. Off. EU: Luxembourg, 2016; pp. e01d8cf+.
